# An Ab Initio Study of Vacancies in Disordered Magnetic Systems: A Case Study of Fe-Rich Fe-Al Phases

**DOI:** 10.3390/ma12091430

**Published:** 2019-05-02

**Authors:** Ivana Miháliková, Martin Friák, Nikola Koutná, David Holec, Mojmír Šob

**Affiliations:** 1 Institute of Physics of Materials, Czech Academy of Sciences, Žižkova 22, CZ-616 62 Brno, Czech Republic; mihalikova@ipm.cz (I.M.); mojmir@ipm.cz (M.Š.); 2 Department of Condensed Matter Physics, Faculty of Science, Masaryk University, Kotlářská 2, CZ-611 37 Brno, Czech Republic; nikola.koutna@tuwien.ac.at; 3 Institute of Materials Science and Technology, TU Wien, Getreidemarkt 9, A-1060 Vienna, Austria; 4 Department of Materials Science, Montanuniversität Leoben, Franz-Josef-Strasse 18, A-8700 Leoben, Austria; david.holec@unileoben.ac.at; 5 Department of Chemistry, Faculty of Science, Masaryk University, Kotlářská 2, CZ-611 37 Brno, Czech Republic; 6 Central European Institute of Technology, CEITEC MU, Masaryk University, Kamenice 5, CZ-625 00 Brno, Czech Republic

**Keywords:** Fe_3_Al, Fe-Al, vacancies, magnetism, ab initio, disorder, superalloys

## Abstract

We have performed quantum-mechanical calculations to examine the impact of disorder on thermodynamic, structural and electronic (magnetic) properties of Fe-Al systems with vacancies. A series of supercells was used and their properties were computed employing density-functional theory (DFT) as implemented in the VASP package. Our case study is primarily aimed at a disordered solid solution Fe${}_{81.25}$Al${}_{18.75}$ but we have compared our results also with those obtained for the ordered Fe${}_{3}$Al intermetallic compound for which experimental data exist in literature. Both phases are found in Fe-Al-based superalloys. The Fe-18.75at.%Al solid solution was simulated using special quasirandom structures (SQS) in three different disordered states with a different distribution of Al atoms. In particular, we have considered a general disordered case (an A2-like variant), the case without the first nearest neighbor Al-Al pairs (a B2-like distribution of atoms) and also the case without both the first and second nearest neighbor Al-Al pairs (the D0${}_{3}$-like variant, in fact, an Fe-rich Fe${}_{3}$Al phase). The vacancy formation energies as well as the volumes of (fully relaxed) supercells with vacancies showed a large scatter for the disordered systems. The vacancy formation energies decrease with increasing concentration of Al atoms in the first coordination shell around the vacancy (an anti-correlation) for all disordered cases studied. The computed volumes of vacancies were found significantly lower (by 25–60%) when compared with the equilibrium volume of the missing atoms in their elemental states. Lastly, we have analyzed interactions between the vacancies and the Fe atoms and evaluated vacancy-induced changes in local magnetic moments of Fe atoms.

## 1. Introduction

Vacancies are among the most frequently occurring defects in solids. Their concentration and importance grow with increasing temperature, in particular when approaching the melting point. Their higher concentrations can be, nevertheless, found also in samples at low temperatures after quenching higher-temperature states containing higher numbers of vacancies and taking advantage of sluggish kinetics at lower temperatures.

The complexity of vacancy-containing systems increases with increasing number of sublattices (where each of them represents atomic sites with a specific local crystallographic environment), the number of chemical species present in studied materials and, last but not least, other degrees of freedom involved (such as different magnetic states which we consider below). The topic of our current research is the impact of disorder on vacancy-related characteristics in magnetic systems. As far as experimental studies are concerned, positron annihilation is one of methods which is sensitive to vacancies, see, e.g., Refs [1,2,3,4,5,6,7,8,9] but the obtained information is often averaged rather than specific to one type of vacancies. In contrast to that, quantum-mechanical calculations can provide atomic-scale site-specific information about each particular type of vacancies including those in magnetic systems, see, e.g., Refs. [1,2,3,4,5,6,7,10,11,12,13,14,15,16,17,18,19,20,21].

**Figure 1:**
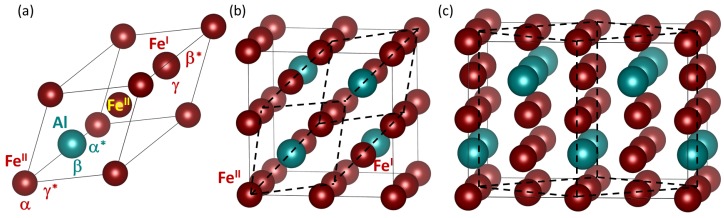
Visualizations of (**a**) a 4-atom primitive cell of Fe${}_{3}$Al, (**b**) a cubic-shape 16-atom elementary supercell of Fe${}_{3}$Al with dashed lines showing schematically crystallographic relation between the 4-atom and 16-atom cells (a face-centered-cubic-like arrangement of 4-atomic primitive cell), and (**c**), a 32-atom $\sqrt{2}\times \sqrt{2}\times 1$ (times 16 atoms) supercell of Fe${}_{3}$Al with dashed lines indicating crystallographic relation between the 16-atom and 32-atom cells. Different sublattices are often denoted by either Greek letters or Arabic or Roman numbers as upper indexes. Regarding the Greek-letter nomenclature, the Fe sublattices are marked as α (Fe sites with 8 Fe atoms in the 1st coordination shell) and γ (Fe sites with 4 Fe and 4 Al atoms in the 1st nearest neighbour shell) and the Al sublattice is denoted by β—see, for instance Refs. [5,6,60]. As far as the upper Roman number indexes are concerned, see, e.g., Ref. [61], they reflect the fact that the Fe${}^{\mathrm{II}}$ sites are twice as abundant as the Fe${}^{I}$ sites. Regarding the upper Arabic number indexes (Fe${}^{1}$ and Fe${}^{2}$), they have the identical meaning reflecting different abundances (see, e.g., Ref. [62]). An alternative use of Greek letters can be found in Ref. [63] where the Al sublattice is denoted α (we show it as $\alpha^{*}$ in part (**a**) in order not to confuse with the other nomenclature), the Fe${}^{I}$ sites is denoted by β (it is visualized as $\beta^{*}$ in part (**a**)) and the Fe${}^{\mathrm{II}}$ sublattice is denoted γ (shown as $\gamma^{*}$).

In our study we focus on magnetic disordered Fe-rich Fe-Al phases. Our choice is motivated by the fact that these materials can have the concentration of quenched-in vacancies as high as a few percent [22,23,24]. Iron-aluminides are interesting for their possible high-temperature applications due to their oxidation resistance [25], stability in molten salts [26], relatively low density, electrical resistivity and low cost of raw materials [27,28,29]. Their wider use is partly hampered by their environmental embrittlement [30,31,32,33] but there is recently a renewed interest in these materials [25,34,35,36,37,38,39,40,41,42,43], including also Fe-Al-based nanocomposites [44,45,46,47,48,49,50,51,52,53,54,55,56,57]. A subset of these nanocomposites is constituted by so-called superalloys consisting of an ordered Fe${}_{3}$Al intermetallic compound with the D0${}_{3}$ structure (see Figure 1) and a disordered solid solution of about 18.75 at.% Al in the Fe matrix, see, e.g., Refs. [46,48,58,59]; we study both below.

## 2. Methods

**Figure 2:**
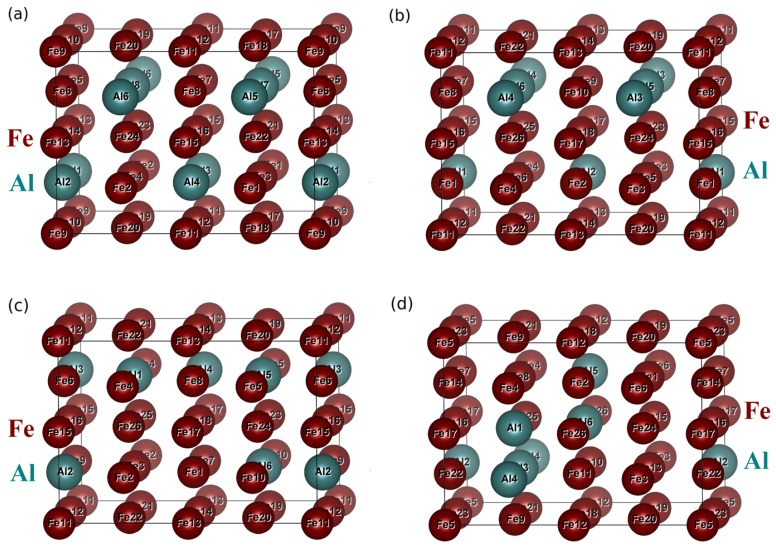
Schematic visualization of 32-atom $\sqrt{2}\times \sqrt{2}\times 1$ (times 16 atoms) supercells used in our ab initio calculations: (**a**) model of ordered Fe${}_{3}$Al compound with the supercell stoichiometry Fe${}_{24}$Al${}_{8}$, (**b**) SQS without the 1st and 2nd nearest neighbour Al-Al pairs (in fact Fe-rich Fe${}_{3}$Al, D0${}_{3}$-like structure) with the supercell stoichiometry Fe${}_{26}$Al${}_{6}$, (**c**) SQS without the 1st nearest neighbour Al-Al pairs (B2-like structure) with the supercell stoichiometry Fe${}_{26}$Al${}_{6}$, (**d**) model of the general disordered phase representing SQS (A2-like structure) with the supercell stoichiometry Fe${}_{26}$Al${}_{6}$. The numbering of individual atoms will be essential for discussion of our computed results below.

Our ab initio calculations were performed within the framework of density functional theory [64,65] with the help of the Vienna Ab initio Simulation Package (VASP) [66,67,68]. The exchange and correlation energy was treated in the generalized gradient approximation (GGA) as parametrized by Perdew and Wang (PW91) [69] using projector augmented wave (PAW) pseudopotentials [70] and the Vosko-Wilk-Nusair correction [71]. We have employed a carefully chosen parametrization (Perdew-Wang, PW91) of the generalized gradient approximation (GGA) and a computational set-up which ensures that the experimental D0${}_{3}$ ground-state structure of Fe${}_{3}$Al is reproduced. Based on convergence tests, we used a plane-wave energy cut-off of 350 eV and a 6 × 6 × 10 Monkhorst-Pack [72] k-point mesh in the case of 32-atom $\sqrt{2}\times \sqrt{2}\times 1$ (times 16 atoms) supercells for calculations of properties of individual phases (see Figure 1c and Figure 2a–d). Our calculations discussed below simulate the vacancy concentration of 3.125% and periodic boundary conditions apply. Regarding ${\mathrm{Fe}}_{3}\mathrm{Al}$ intermetallic compound, two crystallographically different atomic sites for Fe atoms exist: ${\mathrm{Fe}}^{I}$ atoms (γ sublattice) and ${\mathrm{Fe}}^{\mathrm{II}}$ atoms (α sublattice), see Figure 1a. The ${\mathrm{Fe}}^{\mathrm{II}}$ atoms are surrounded by 4 ${\mathrm{Fe}}^{I}$ atoms and 4 Al atoms (β sublattice) and each of these two 4-atom neighbor groups form a tetrahedron with atoms being mutually all the 3rd nearest neighbors. The ordered intermetallic compound Fe${}_{3}$Al is also visualized using a 32-atom supercell (Figure 1c) with the composition Fe${}_{24}$Al${}_{8}$. A crystallographic relation of this 32-atom representation of Fe${}_{3}$Al and its cubic-shape 16-atom cell is schematically shown in Figure 1c by the dashed lines. The 32-atom supercell of Fe${}_{3}$Al is then visualized also in Figure 2a with the individual atoms numbered.

The numbering allows to put three supercells representing three disordered Fe-18.75 at.% Al states into a crystallographic context of Fe${}_{3}$Al compound, see Figure 2b–d. When designing the models of the disordered Fe${}_{81.25}$Al${}_{18.75}$ phase we used a concept of special quasi-random structure (SQS) [73] generated by USPEX code [74,75,76]. The used 32-atom supercells (Figure 2b–d) offer a wide range of distributions of aluminium atoms in the disordered Fe-Al phase and also a higher number of non-equivalent atomic positions. These three models for the disordered Fe-18.75 at.% Al states contain the Fe and Al atoms according to the formula Fe${}_{26}$Al${}_{6}$.

In order to examine the energy cost of a vacancy introduction in a material (the vacancy formation energy, $E_{f}$) we employ the formula

(1) $$E_{f}=E_{\mathrm{def}}-E_{\mathrm{perf}}+\sum_{x}n_{x}^{\mathrm{vac}}\;E_{x},$$

where $E_{\mathrm{def}}$ is total energy of the defected system, $E_{\mathrm{perf}}$ is total energy of the vacancy-free system, $n_{x}^{\mathrm{vac}}$ represents the number of missing atoms in the supercells and $E_{x}$ is their chemical potential (bcc ferromagnetic Fe and fcc non-magnetic Al).

## 3. Results

Before we proceed with calculations of vacancies in disordered Fe-Al states, which are the main topic of our study, we first test our methodology in the case of the Fe${}_{3}$Al intermetallic compound, which has been intensively studied in the past both theoretically and experimentally. Our computed vacancy formation energies for all three possible types of vacancies are summarized in Table 1 together with other computational as well as experimental data.

Our theoretically obtained vacancy formation energies $E_{f}$ in the case of the Fe${}_{3}$Al compound are in a very good agreement with the previous theoretical values reported by Kuriplach [6] and Deniszczyk et al. [7] employing generalized gradient approximation (GGA). On the other hand, the vacancy formation energies calculated by other groups using local density approximation (LDA) are not only quantitatively but also qualitatively different. In particular, according to Fähnle et al. [60] and Mayer et al. [5] the Al vacancy formation energies are lower than those of the Fe vacancies on the γ sublattice, while we and the other GGA calculations [6,7] predict the opposite relation. Unfortunately, experimental data of Fe vacancy formation energies by Wolff et al. acquired by Doppler broadening (DB) and positron lifetime measurements (LT) are not compared with values for the Al sublattice in these two studies. Moreover, in the study by Wolff et al. [77] it was not specified from which sublattice the missing Fe atoms are. According to our calculated values of vacancy formation energies, it may be deduced that vacancies studied in those papers are formed on the α sublattice as these are the easiest defects to be created.

After successfully testing our methodology, as a next step, we considered vacancies in the disordered Fe-Al systems. Here the vacancy formation energies are found sensitive to the chemical composition and distribution of atoms around the vacancy. Consequently, ranges of vacancy formation energies are obtained instead of a single value for each sublattice as it was the case in the Fe${}_{3}$Al. Considering the most disordered Fe${}_{81.25}$Al${}_{18.75}$ phase modeled by a fully disordered SQS structure (the A2-like variant shown in Figure 2d), the formation energies of Fe vacancies span from 1.601 to 2.232 eV and those of Al vacancies are predicted to be from 2.118 to 2.751 eV. As we are not aware of any experimental vacancy formation energies for the simulated composition (Fe${}_{81.25}$Al${}_{18.75}$), a comparison with experimental data can be only indirect. Focusing on the energies of Fe vacancies, Schaefer et al. [61] investigated the formation of Fe vacancies in Fe${}_{76.3}$Al${}_{23.7}$ using the positron lifetime measurements and their detected value, 1.18 eV, is not very far from the lower bound of our theoretical values, i.e., 1.601 eV. Under other conditions, Wolff et al. examined Fe${}_{93}$Al${}_{7}$ (i.e., a lower Al concentration) by Doppler broadening measurement and reported the value of 1.37 eV. Therefore, both the experiments and our calculations show a qualitatively similar trend: the vacancy formation energies grow when lowering the concentration of Al.

**Figure 3:**
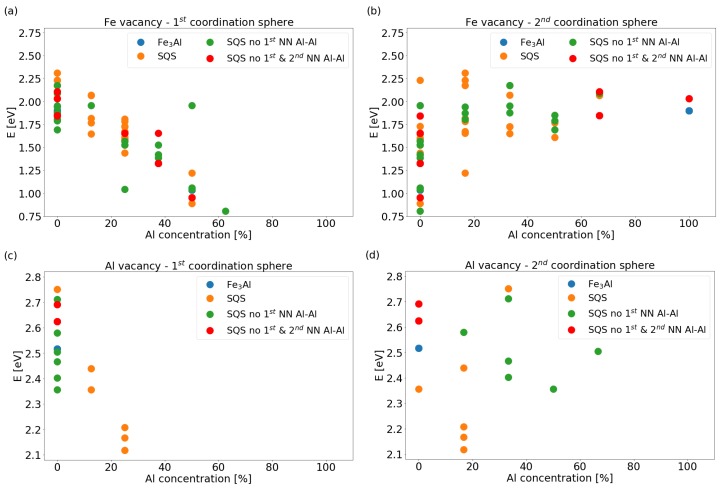
Vacancy formation energies in the studied phases as functions of concentration of Al atoms in the coordination shells around the removed atom: for the Fe vacancy (**a**) in the 1st (**b**) and the 2nd coordination shell, and for Al vacancy (**c**) in the 1st (**d**) and the 2nd shell.

The above identified tendency is further examined by analyzing the calculated formation energies $E_{f}$ of Fe and Al vacancies as functions of concentrations of Al atoms found in the first and second coordination sphere around the vacancy. The results are shown in Figure 3 and reveal interesting trends: the vacancy formation energy decreases with increasing concentration of Al atoms in the 1st coordination shell for both Fe and Al vacancies, see Figure 3a,c. Regarding the concentration of Al atoms in the 2nd coordination shells (see Figure 3b,d), the trends are much less pronounced and much more difficult to be identified. The Fe vacancy formation energies seem to sharply increase for the Al concentration between 0 at.% and 16.67 at.% (1 out of 6) and then stay rather constant with a weak wavy pattern (see Figure 3b). The tendencies in the data points related to the Al vacancy formation energies as a function of the Al concentration in 2nd coordination shell around vacancies (see Figure 3d) are even less pronounced. All these results nevertheless illustrate a remarkable sensitivity of vacancy formation energies to the distribution and chemical composition of atoms in their neighboring sites.

The results in Figure 3 were obtained when fully adapting the volume and supercell shape to the presence of a vacancy by minimizing the energy. This approach corresponds to the situation when the matrix surrounding the vacancies is soft enough to adapt in this way and as a model it corresponds to the rather high simulated vacancy concentration. As another extreme we can imagine a scenario when the concentration of vacancies is very low and a large defect-free matrix further apart from a vacancy would be hard enough to keep the interatomic distances and volume as in the defect-free state. For comparison, we have calculated the vacancy formation energies of Fe atoms also for the latter scenario and our results are shown in Appendix A. Importantly, the above discussed trends are the same.

**Figure 4:**
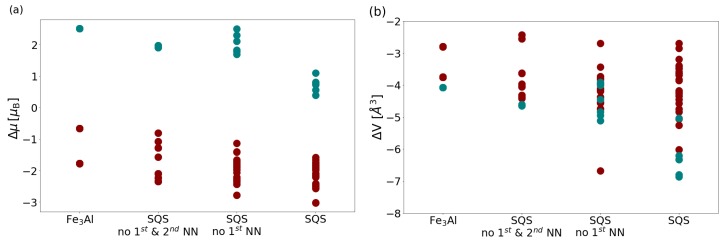
Computed changes of the overall magnetic moment (**a**) and volume (**b**) of supercells shown in Figure 3a–d. The data points are organized in vertical columns for each calculated supercell.

Next to the energies, our calculations also allow for determining the changes of the overall volume and the total magnetic moment of the studied systems, i.e., the supercells used in our calculations. Our results related to the overall volume and the magnetic moment of the supercells are summarized in Figure 4a,b, respectively. The results obtained for the three simulated disordered states are compared with those for the ordered Fe${}_{3}$Al intermetallic compound. The values of the total magnetic moments and the volume for each simulated system without vacancies are taken as references. The results shown in Figure 4 are then evaluated so that these references are subtracted from the values obtained for vacancy-containing supercells. Focusing first on the vacancy-induced changes in the total magnetic moments in Figure 4a, it is possible to see a trend when a removal of Al atoms in any of the four studied systems (full blue circles in Figure 4a) leads to an increase of the total magnetic moment. This impact is most visible in the case of the ordered Fe${}_{3}$Al intermetallic compound (the left-most column of data points in Figure 4a). This finding is in agreement with the lowering of magnetic moment of the Fe atoms with increasing number of Al atoms in the first coordination shell which was theoretically identified earlier in Ref. [78] or in our recent publications [79,80]. In an opposite manner, a removal of Fe atoms typically leads to a lowering of the total magnetic moment (full red-brown circles in Figure 4a). Here the lowering is smaller in the case of Fe${}_{3}$Al and more significant for the computed disordered systems. The maximum reduction of the total magnetic moments (the most negative values in Figure 4a) are those computed for vacancies in the disordered systems.

As far as the volumetric changes are concerned, see Figure 4b, the minimum volume reduction is predicted for Fe${}^{I}$-like atoms in the disordered supercell without any first and second nearest neighbor Al-Al pairs, the D0${}_{3}$-like case, i.e., the Fe-rich Fe${}_{3}$Al and in the case of Fe${}^{I}$ atoms (γ sublattice) in the Fe${}_{3}$Al. The other extreme, the maximum volumetric reduction is predicted for Al vacancies in a general SQS with a disorder in all sublattices, the A2-like state (full blue circles in the right-most column of data points in Figure 4b). Next, we will compare the volumetric changes induced by the missing atoms with the equilibrium volume of these absent atoms in their elemental phases.

**Figure 5:**
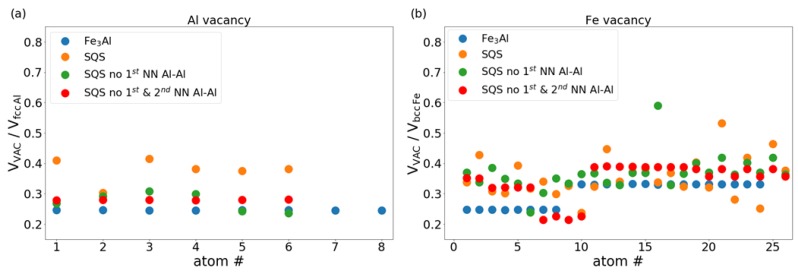
Computed volumes of Al (**a**) and Fe (**b**) vacancies expressed relatively with respect to the equilibrium volume of Al atom in the non-magnetic face-centered-cubic (fcc) Al and ferromagnetic body-centered-cubic (bcc) Fe, respectively.

**Figure 6:**
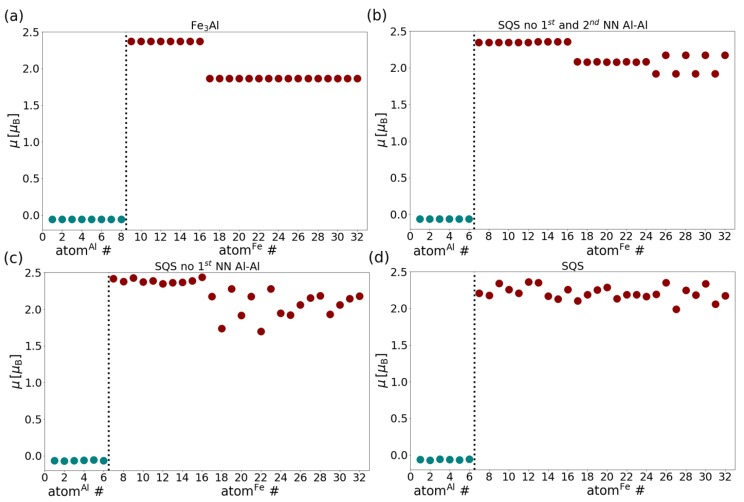
Dependencies of local magnetic moments on numbers of individual atoms in all studied phases: (**a**) stoichiometry Fe${}_{24}$Al${}_{8}$ - 8 Al atoms with numbers from 1 to 8, 8 Fe${}^{I}$ atoms (sublattice γ) with numbers from 9 to 16 and 16 Fe${}^{\mathrm{II}}$ atoms (sublattice α) with numbers from 16 to 32, (**b**–**d**) stoichiometry Fe${}_{26}$Al${}_{6}$-6 Al atoms with numbers from 1 to 6 and 26 Fe atoms with numbers from 7 to 32 (data points for Al and Fe atoms are separated by vertical dotted lines).

Figure 5 summarizes our results with the calculated volumes of the Al and Fe vacancies visualized relatively to the equilibrium volume of the non-magnetic face-centered-cubic (fcc) Al and ferromagnetic body-centered-cubic (bcc) Fe, respectively. The volumes of Al vacancies are between 25% and 42% of the equilibrium volume of non-magnetic fcc Al. The volumes of Fe vacancies span over a yet broader range with the lower bound very similar as in the Al case but the upper one is close to 60% of the equilibrium volume of FM bcc Fe. Next we will analyze the sensitivity of local atomic moments to the chemical composition of the neighborhoodand distribution of atoms. In order to examine the changes induced by the presence of vacancies, we first show the local magnetic moments of atoms in vacancy-free supercells - their values are displayed in Figure 6.

In the case of the ordered Fe${}_{3}$Al intermetallic compound (Figure 6a), the local magnetic moments of Al atoms are almost zero (−0.057 $\mu_{B}$) and the Fe${}^{I}$ atoms on the sublattice γ have a higher magnitude of the local magnetic moment (2.374 $\mu_{B}$) than Fe${}^{\mathrm{II}}$ atoms on the sublattice α (1.866 $\mu_{B}$). This is in agreement with results of previous theoretical studies, see e.g., Ref. [78], which showed that the magnitude of the local magnetic moment of Fe atoms decreases with increasing concentration of Al atoms in the first coordination sphere. Here the Fe${}^{I}$ atoms with a higher magnetic moment have 8 Fe atoms in their first coordination sphere while the Fe${}^{\mathrm{II}}$ atoms with a lower magnetic moment have only 4 Fe atoms and 4 Al atoms in the first coordination shell. In cases of disordered Fe-Al models (Figure 6b–d), it is difficult to determine a clear pattern. While Al atoms exhibit almost zero values of magnetic moments (from −0.072 to −0.056 $\mu_{B}$), Fe atoms possess local magnetic moments from a wide range of values (from 1.696 $\mu_{B}$ to 2.437 $\mu_{B}$). The disorder, which involves different local atomic environments of many atoms, lifts the degeneracy. Therefore, the local magnetic moments cover a range of values rather than a single value. Interestingly, the scatter of these values of local magnetic moments are not simply correlated with the level of simulated disorder. In particular, the maximum scatter of values is visible in Figure 6c which corresponds to the situation when there are no first-nearest neighbor Al-Al pairs (B2-like distribution of atoms) and the A2-like disordered case (Figure 6d) is characterized by a narrower range of values. A possible explanation can be that all Fe atoms obtain rather similar disordered environment, which does differ for each atom but there are no longer any sublattices which offer specific surroundings (and thus values of local magnetic moments which are clearly different from more disordered sites).

**Figure 7:**
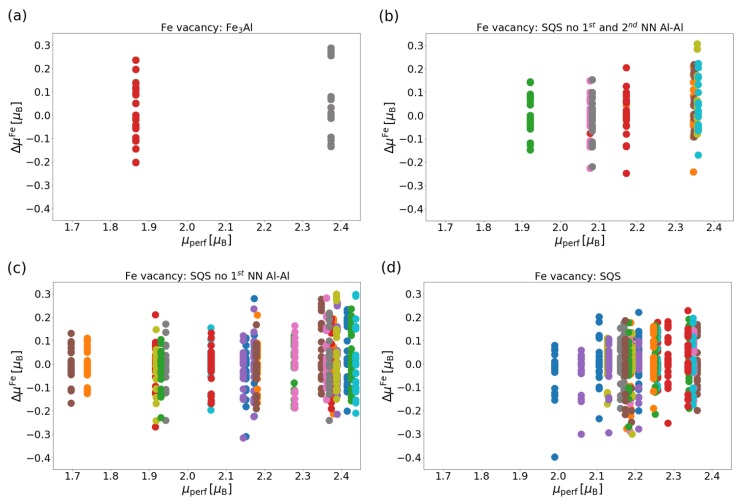
Change in local magnetic moments $\Delta \mu^{\mathrm{Fe}}$ as a function of local magnetic moments of atoms in vacancy-free supercells: (**a**) ordered Fe${}_{3}$Al intermetallic compound, (**b**) SQS without the 1st and 2nd nearest neighbour Al-Al pairs (in fact Fe-rich Fe${}_{3}$Al), (**c**) SQS without the 1st nearest neighbour Al-Al pairs (B2-like structure), (**d**) a fully disordered phase SQS (A2-like structure).

Having discussed the local magnetic moments of atoms in vacancy-free cases, we proceed to vacancy-containing states and vacancy-induced changes of local magnetic moments at atoms around simulated vacancies. In particular, we evaluate dependencies of changes in local magnetic moments ($\Delta \mu^{\mathrm{Fe}}=\mu_{\mathrm{vac}}^{\mathrm{Fe}}-\mu_{\mathrm{perf}}^{\mathrm{Fe}}$) on individual atoms in all studied phases. As we systematically take away one Fe atom at a time, one Fe atom after another, we obtain for each Fe atom up to 25 different values of its local magnetic moment corresponding to the case when one of the other 25 atoms (often in a different distance and in a different crystallographic direction) is removed. Figure 7 summarizes our results, i.e., the vacancy-induced changes of the local magnetic moments as a function of the local magnetic moment of the monitored Fe atom in the vacancy-free state (see the values in Figure 6).

Discussing the ordered Fe${}_{3}$Al case first, there are two different values of local magnetic moments of Fe atoms on different sublattices shown (see Figure 6a) and, therefore, there are two vertical columns of data points showing changes of the local magnetic moment of these two different types of Fe atoms when different other atoms are removed. Figure 7a shows that the vacancy-induced changes are both negative and positive and within the range from −0.2 to 0.3 $\mu_{B}$.

When focusing on the Fe-18.75 at.%Al disordered system, Figure 7 neatly shows how the increasing level of disorder leads to a higher number of different local magnetic moments in the vacancy-free case (corresponding to a higher number of vertical columns of data points representing the changes in the local magnetic moment) and how differently these crystallographically non-equivalent Fe atoms respond to the presence of Fe vacancies in different distances and different crystallographic directions. Here we limit ourselves to Fe vacancies only as they have lower formation energies and are more probable to occur. The visualization in Figure 7 shows that the vacancy-induced changes in local magnetic moments of Fe atoms are within a fairly wide range from −0.4 to 0.3 $\mu_{B}$.

**Figure 8:**
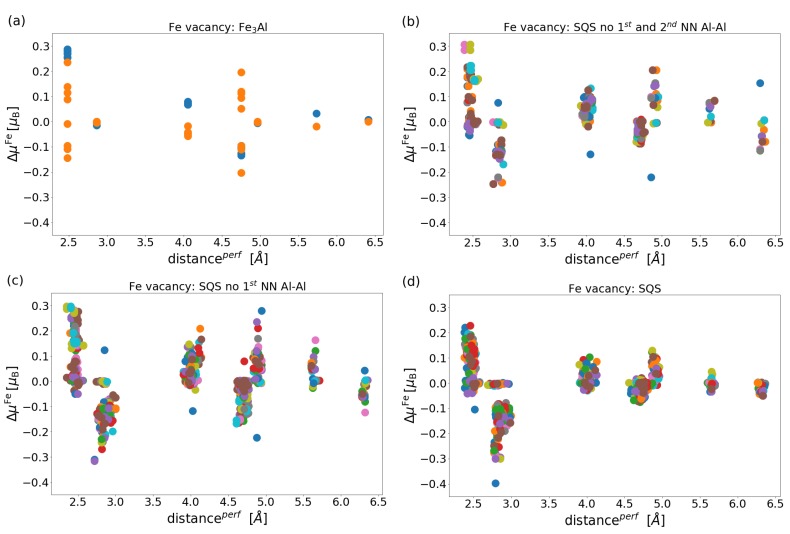
Change in local magnetic moments of the Fe atoms $\Delta \mu^{\mathrm{Fe}}$ as a function of the distance of studied atoms from the vacancy (measured in the vacancy-free states): (**a**) ordered Fe${}_{3}$Al intermetallic compound, (**b**) SQS without the 1st and 2nd nearest neighbour Al-Al pairs, (**c**) SQS without the 1st nearest neighbour Al-Al pairs (B2-like structure), (**d**) a general disordered phase SQS (A2-like structure).

While Figure 7 shows the magnitude of vacancy-induced changes of the local magnetic moment of atoms which remain in the material (vertical axis) as a function of the local magnetic moment which the removed atom has in a vacancy-free material (horizontal axis), it does not provide information about how these changes depend on the distance of the Fe atoms from the vacancy. Motivated by the dependencies published in the case of interstitial atoms (see, e.g., Ref. [81]), we visualize these trends in Figure 8. Besides the magnitude of changes of the local magnetic moments (vertical scatter of data points) the Figure 8 neatly illustrates also the variations of inter-atomic distances in the vacancy-free states due to the disordered distribution of atoms (horizontal scatter of data points).

As seen in Figure 8, the changes of the local magnetic moments clearly decrease with the increasing distance from the vacancy only in the case of the general SQS, see Figure 8d. As far as the other states are concerned, see Figure 8a–c, the connection between the vacancy-induced changes and the distance from it are much less pronounced. This finding can indicate a complex interplay of changes of the local magnetic moments via rather long-distance interactions and coupling of different (structural, compositional, electronic, magnetic and vibrational) degrees of freedom.

Lastly, when considering methodological aspects, it should be stated that our use of special quasi-random structures when modeling vacancies is only one of a few alternatives. The other options include the application of Monte Carlo simulations, see, e.g., Refs. [82,83] or the use of cluster expansion technique, see, for example, Refs. [84,85]. As another limitation of our approach it is worth mentioning that the vacancies change the pair-distribution functions originally evaluated when generating the vacancy-free supercells (see them in Figure 2). Consequently, there are subtle and complex consequences as far as thermodynamic and statistical aspects of the studied systems are concerned. For a proper approach to these aspects see, for example, a recent publication [86]. These will be the topic of future studies.

## 4. Conclusions

We have performed a series of quantum-mechanical calculations to analyze thermodynamic, structural and magnetic properties of two Fe-Al phases with vacancies. Our computational set-up and, in particular, the parametrization (Perdew-Wang, PW91) of the generalized gradient approximation (GGA), were carefully chosen so as to ensure that the experimental D0${}_{3}$ ground-state structure of Fe${}_{3}$Al is reproduced. Our calculated vacancy formation energies in the case of ordered Fe${}_{3}$Al intermetallic compound (1.04 eV, 1.90 eV and 2.52 eV for the α-sublattice Fe, γ-sublattice Fe and Al vacancies, respectively), are in agreement with both previous theoretical GGA values and published experimental data (0.92–1.08 eV) for Fe vacancies at the α-sublattice. The Fe${}_{3}$Al case was studied as a test of our methodology but we were primarily interested in a disordered solid solution Fe${}_{81.25}$Al${}_{18.75}$. To simulate it we used special quasirandom structures (SQS) to model three different disordered states with different distribution of Al atoms in this material. In particular, we have considered a general disordered case without any sublattices (an A2-like variant), the distribution of atoms without the first nearest neighbor Al-Al pairs (a B2-like case) and also the case when both the first and second nearest neighbor Al-Al pairs are eliminated (the D0${}_{3}$-like variant of an Fe-rich Fe${}_{3}$Al).

As a main conclusion of our study, a remarkable sensitivity of vacancy formation energies, their volumes and changes of local magnetic moments of neighboring Fe atoms on the chemical composition around the vacancy was found. The vacancy formation energies as well as the volumes of (fully relaxed) supercells with vacancies showed a large scatter for the disordered systems. They mostly decrease with increasing concentration of Al atoms in the first coordination shell around the vacancy for all calculated disordered cases. Dependencies on the chemical and structural distribution of atoms in the second shell are less clear but nearly opposite trends (increasing and nearly constants formation energies as a function of the concentration of Al atoms) neatly illustrate the complexity of vacancies in disordered systems.

The volumes of vacancies were found significantly lower (by as much as 75%) when compared with the volumes of the missing atoms in their elemental forms. Finally, we have analyzed interactions between the vacancies and the Fe atoms and evaluated vacancy-induced changes in local magnetic moments of Fe atoms. These changes of the local atomic moments were found within a range from −0.4 to 0.3 $\mu_{B}$.

Considering how rich and complex are the phenomena related to the vacancies in disordered magnetic systems, we hope that our work will motivate future studies in this area (see, for example, recent works in Refs. [20,21]). Not only experimental but also theoretical studies would be highly desirable as quantum-mechanical calculations can provide reliable values of local magnetic moments, volumes and energies with atomic resolution, i.e., characteristics of vacancies which are experimentally available often only in an averaged manner. Moreover, it would be highly advantageous to apply in these disordered cases a suitable method, based on thermodynamics and statistical physics, which estimates temperature-dependent concentration of vacancies. In contrast to the case of vacancies in ordered intermetallics, which are characterized by a few individual values, broad ranges of vacancy formation energies (or better said, their statistical distributions) would be used as input.

## Figures and Tables

**Table 1:** Vacancy formation energies of Fe${}_{3}$Al compound (with 3.125 at.% vacancies). Theoretical values were reported in the following studies: Kuriplach [6], Deniszczyk et al. [7], Fähnle et al. [60], Muratov et al. [63], Mayer et al. [5]. Experimental values of vacancy formation energies were obtained by Doppler broadening (DB) and positron lifetime measurements (LT) as reported by Wolff et al. [77].

Reference	This Work	[6]	[7]	[60]	[63]	[5]	[77]	[77]
**Method**	**GGA PW91**	**GGA PW91**	**GGA PBE96**	**LDA**	**LDA**	**LDA**	**DB**	**LT**
E${}_{f}$ (Fe α) [eV]	1.04	0.88	1.19	1.18	1.26	1.25	1.08	0.92
E${}_{f}$ (Fe γ) [eV]	1.90	1.70	1.90	2.45	2.36	2.27		
E${}_{f}$ (Al β) [eV]	2.52	1.85	2.50	1.53	3.23	1.38		

**Figure A1:**
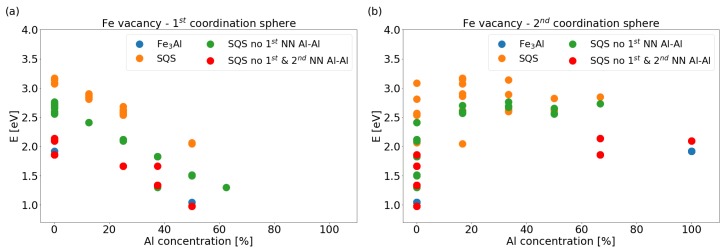
Vacancy formation energies calculated without changes in cell shape and volume as functions of concentration of Al atoms: for Fe vacancy (**a**) in the 1st (**b**) and the 2nd coordination shell.

## Appendix A

Our above discussed quantum-mechanical calculations of the vacancy formation energies and vacancy-related properties were all performed in a way that the shape and volume of supercells containing defects were changed so as to minimize the total energy. This situation corresponds to a rather high concentration of vacancies and the surrounding matrix which is soft enough to respond to the vacancies. As a complement to these calculations we also test a scenario when the concentration of vacancies is very low and a large defect-free matrix further apart from a vacancy would be hard enough to keep the interatomic distances and volume as in the defect-free state. Methodologically, this case corresponds to the situation when the volume and the shape of supercells with vacancies are kept equal to those of the defect-free material. Only the internal atomic positions are allowed to change so as to minimize the forces acting upon them (and the total energy). The calculated vacancy formation energies are shown in Figure A1. The formation energies are higher than those summarized in Figure 3, but the above identified trend of decreasing vacancy formation with increasing concentration of Al atoms in the 1st coordination shell is clearly visible also in this case (see Figure A1a).
